# Profiling the Quality and Quantity of Naturally Induced Antibody Responses Against Pfs230 and Pfs48/45 Among Non-Febrile Children Living in Southern Ghana: A Longitudinal Study

**DOI:** 10.3389/fcimb.2021.770821

**Published:** 2021-11-25

**Authors:** Fermin K. Broni, Festus K. Acquah, Dorcas Obiri-Yeboah, Evans K. Obboh, Esther Sarpong, Linda E. Amoah

**Affiliations:** ^1^ Department of Microbiology and Immunology, School of Medical Sciences, University of Cape Coast, Cape Coast, Ghana; ^2^ Immunology Department, Noguchi Memorial Institute for Medical Research, University of Ghana, Accra, Ghana; ^3^ West African Centre for Cell Biology of Infectious Pathogens, University of Ghana, Accra, Ghana; ^4^ Directorate of Research, Innovation and Consultancy, University of Cape Coast, Cape Coast, Ghana; ^5^ Department of Molecular Biology and Biotechnology, School of Biological Sciences, University of Cape Coast, Cape Coast, Ghana

**Keywords:** antibody levels, relative avidity, gametocyte, *P. falciparum*, polymerase chain reaction (PCR)

## Abstract

A clear understanding of the properties of naturally induced antibody responses against transmission-blocking vaccine candidates can accelerate the understanding of the development of transmission-blocking immunity. This study characterized the naturally induced IgG responses against two leading transmission-blocking vaccine antigens, Pfs230 and Pfs48/45, in non-febrile children living in Simiw, Ghana. Consecutive sampling was used to recruit 84 non-febrile children aged from 6 to 12 years old into the 6-month (November 2017 until May 2018) longitudinal study. Venous blood (1 ml) was collected once every 2 months and used to determine hemoglobin levels, *P. falciparum* prevalence using microscopy and polymerase chain reaction, and the levels and relative avidity of IgG responses against Pfs230 and Pfs48/45 using indirect ELISA. IgG levels against Pfs230 and Pfs48/45 decreased from the start (November) to the middle (January) and end (March) of the dry season respectively, then they began to increase. Participants, especially older children (10–12 years old) with active infections generally had lower antibody levels against both antigens. The relative avidities of IgG against both antigens followed the trend of IgG levels until the middle of the dry season, after which the relative avidities of both antigens correlated inversely with the antibody levels. In conclusion, although IgG antibody levels against both Pfs48/45 and Pfs230 began to increase by the early rainy season, they were inversely correlated to their respective relative avidities.

## Introduction

The cycle of malaria transmission can be broken by the development of an immune response that arrests the progress of the infectious stages of the parasite, including gametocytes. *Plasmodium falciparum* antigens Pfs230 and Pfs48/45 are among the most widely characterized gametocyte surface proteins (Arredondo et al., 2012; Jones et al., 2015; MacDonald et al., 2016; Acquah et al., 2017). The Pfs230 is a 3,135 amino acid (aa) protein containing 14 s48/45 6-Cys domains (Williamson & Kaslow, 1993) and Pfs48/45 is a 448 aa protein containing 3 s48/45 6-Cys domain (Outchkourov et al., 2008). Antibodies against both Pfs230 and Pfs48/45 antigens possess transmission-reducing activities and are thus able to prevent the completion of the sporogonic life cycle of the malaria parasite within the mosquito vector (Kumar et al., 1995; Bousema et al., 2006). Enhanced knowledge of the properties of naturally induced antibody responses against these antigens can help accelerate the understanding of malaria transmission-blocking vaccine (MTBV) candidate development. Children have been identified to efficiently harbor gametocytes (Lamptey et al., 2018) and make them a relevant group to study the quality (avidity) and quantity (level) of naturally induced IgG responses against gametocyte antigens.

Although there are a number of different antibody types, IgG and IgM occurred to be the most commonly examined types (Leoratti et al., 2008; Zakeri et al., 2011; Mayor et al., 2018) which have also been found to keep a *P. falciparum* infection under control (Dodoo et al., 2008). Usually, an encounter between a B cell and parasite antigen activates the B cell with the help of T cell, to proliferate and differentiate into plasma cells. Plasma cells then release IgM primarily and IgG secondarily into circulation (Alam et al., 2013), binding to the target antigen. In many instances, repeated exposure of a B cell to the same antigen results in an increase in the total bond strength of the antibody-antigen complex (avidity) (Howitt et al., 2009; Murugan et al., 2018).

Protection against *P. falciparum* parasites is known to be associated with high antibody avidity rather than just high antibody levels (Bachmann et al., 1997; Ross et al., 2001). However, in some instances, high antibody levels have been found to compensate for low antibody avidity and maintain an individual’s acquired immunity (Ssewanyana et al., 2017). The immunity that results from repeated exposure to the sexual stages of the malaria parasite (malaria transmission-blocking immunity) can prevent the mosquito from becoming infectious and thus reduce malaria transmission rather than protect the individual directly (Healer et al., 1999; White et al., 2018).

This study sought to characterize the properties (levels and relative avidities) of naturally-induced antibody responses against two transmission-blocking vaccine candidates, Pfs230 and Pfs48/45 among non-febrile children in southern Ghana.

## Methods

### Ethics, Study Site, Population, and Sampling

Ethical clearance for this study (NMIR-IRB CPN 024/14-15) was obtained from the Institutional Review Board (IRB) of Noguchi Memorial Institute for Medical Research (NMIMR). Written informed consent was obtained before enrolling participants for this study.

**Figure 1:**
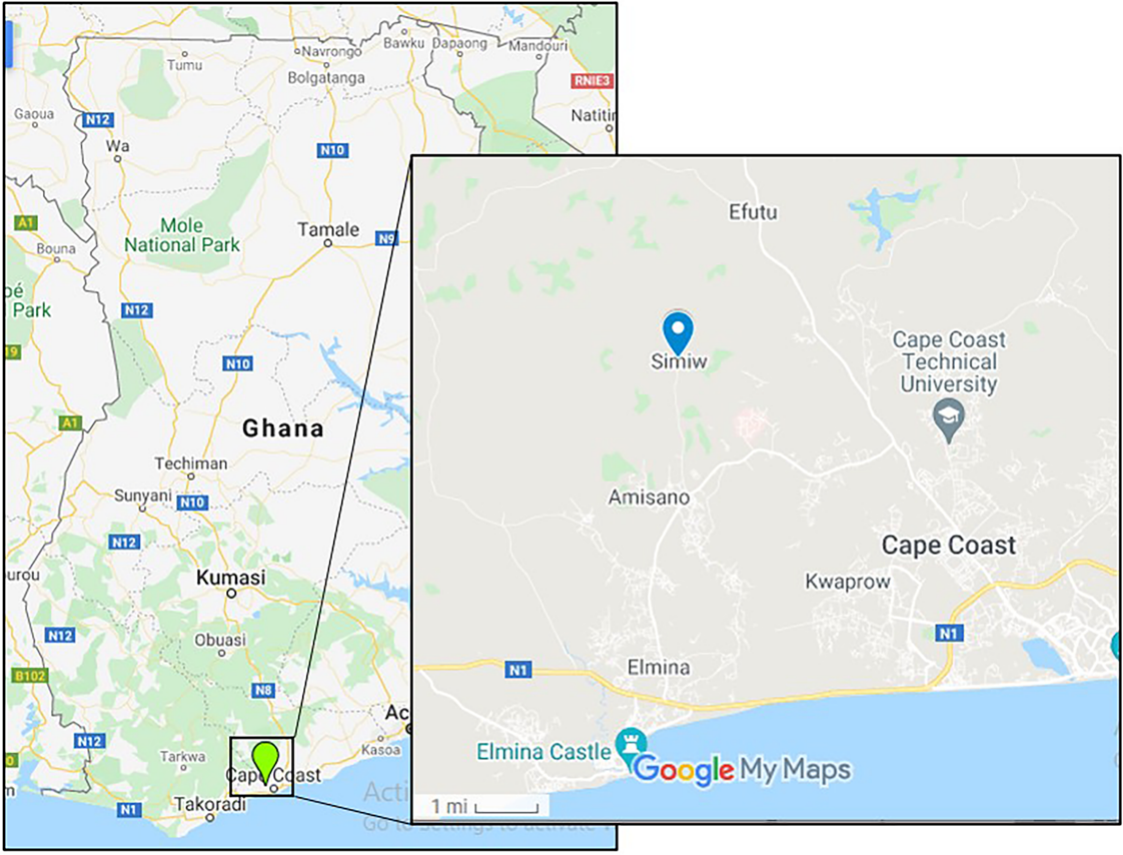
A map of Ghana showing the study site (Simiw) in the enlarged area. The map was generated using Google maps.

The study was conducted in Simiw (**Figure 1**); a peri-urban community in the Komenda–Edina–Eguafo–Abrem (KEEA) Municipal Assembly of the Central Region of Ghana. Malaria transmission in Simiw is usually perennial and overlaps with the rainy season peaking in-between May and July. The dry season however begins in November and ends in April (Acquah et al., 2020). Study participants were pupils of the Simiw M/A Basic School-aged from 6 to 12 years.

A total volume of 1 ml of the venous blood sample was collected from each of the 84 study participants once every 2 months beginning November 2017 until May 2018. Prior to sample collection, the axillary temperature was measured using a digital thermometer. Thick and thin smears were prepared according to WHO guidelines (Organization, 2016). Hemoglobin levels were determined using a drop of whole blood (15 μl) and the Urit 12 HB meter (URIT Medical Electronic Co. Ltd., China). Three 50 μl drops of whole blood were also spotted onto Whatmann #3 filter paper (GE Healthcare, USA). The remaining blood was separated into plasma and packed cells and each stored at −20°C.

### Estimation of Parasite Density

The thick and thin blood smears were processed according to WHO guidelines (Organization, 2016) and as previously described (Amoah et al., 2019b). The smears were observed under 100× oil immersion objectives by two independent trained microscopists and any inconsistency in deciding a positive or negative slide was settled by the reading of a third microscopist. Parasite density was estimated by multiplying the number of parasites counted per 200 white blood cells (WBCs) by 40 based on the assumption that 1 μl of blood contains 8,000 WBCs.

### DNA Extraction

The Saponin/Chelex DNA extraction method was used as previously described (Amoah et al., 2018). Briefly, two 3 mm discs were punched out of the dried filter paper blood spot into a 1.5 ml Eppendorf tube. A measure of 1 ml solution of 1× phosphate-buffered saline and 50 µl of 10% saponin was added to each tube, vortexed, and kept overnight at 4°C. The discs were washed twice with 1 ml phosphate-buffered saline and kept in 30 µl of 20% chelex and 70 µl of Dnase/Rnase water was incubated at 95°C for 10 min with intermittent vortexing. Finally, the tubes were spun for 6 min at 13,000 rpm and the supernatant containing the extracted gDNA was transferred into a sterile 0.5 ml microfuge tube and preserved at −20°C.

### Determination of *P. falciparum* by Polymerase Chain Reaction

Nested polymerase chain reaction, was used to amplify the 18S *rRNA* gene of *P. falciparum* as previously described (Amoah et al., 2019b). The Nest 1 reaction mixture contained 80 nM of primers (rPLU5/rPLU6), 5 μl DNA template, 1× polymerase chain reaction buffer, 167 nM dNTPs, 2.5 mM MgCl_2_, and 1 U of OneTaq DNA polymerase, which totaled up to 15 μl mixture. The nest 2 reaction mixture contained 133.33 nM of primers (rFAL1/rFAL2) and 0.5 μl of the nest 1 product. The cycling conditions were set as follows; initial denaturation (95°C for 5 min), then 35 cycles of denaturation at 94°C for 30 s, annealing at 55 and 58°C (for nest 1 and nest 2 respectively) for 1 min and an extension at 68°C for 1 min with a final extension at 68°C for 5 min. The nest 2 polymerase chain reaction products were gel electrophoresed and visualized using UV illumination.

### Determination of IgG Levels by Indirect Enzyme-Linked Immuno-Sorbent Assay (ELISA)

The IgG levels were determined according to previously described protocols (Amoah et al., 2019a). The Pfs230 antigen used in this study was the prodomain, amino acid (aa) residues 443 to 590 while the Pfs48/45 antigen comprised of the C-terminal 6C region (aa 291–428); both of which were expressed in *Lactococcus lactis* (Acquah et al., 2017). The negative control samples comprised of pooled plasma from malaria naïve individuals. The plasma used for plotting the standard curve and the positive control sample comprised of pooled plasma from individuals who were previously identified as containing high levels of IgG against these two antigens (Arredondo et al., 2012; Jones et al., 2015; MacDonald et al., 2016; Acquah et al., 2017). The Maxisorp plates (Nunc, Thermo Fisher) were coated with 100 µl of antigen (Pfs48/45 or Pfs230) diluted to 1 µg/ml in carbonated buffer (pH of 9.2) and the plasma samples were diluted 1:200 in 1% skimmed milk in 1× PBS containing 0.5% Tween 20. The rest of the procedure was followed through to the reading of the plates using the ELx808 plate reader (BioTek) set at 450 nm as previously described (Amoah et al., 2019a).

### Determination of Relative Avidity by ELISA

The relative avidity of IgG in each sample was determined as previously described (Amoah et al., 2019a). A procedure similar to the ELISA described above was used with an extra step incorporated after the plasma incubation step. Briefly, each plasma sample was added to four wells of the plate, after the set incubation time of 1 h, a 100 μl solution of 2.4 M sodium thiocyanate was added to two of the four wells for 15 min. After this incubation step, the wells of the plates were washed and the procedure followed through with the secondary IgG incubation step to the stop reaction exactly as with the indirect ELISA described above.

### Data Analysis

The OD values obtained from the plate reader were converted into weighted concentrations (wConcs) using ADAMSEL (Ed Remarque, BPRC). Graphs were plotted using GraphPad Prism version 6 and Microsoft Excel. The Kruskal–Wallis test and Dunn’s multiple comparison *post-hoc* tests were used to assess statistical significance between median values of quantitative data between age groups (overall, ≥10 yrs and <10 yrs) and time points (November 2017, January 2018, March 2018 and May 2018). Within-individual variation of the antibody and avidity levels across the study period was assessed with the Friedman test. Association between microscopic and submicroscopic *P. falciparum* prevalence was determined by chi-square test and correlation between IgG responses against Pfs230 and Pfs48/45 were determined using Spearman’s rank correlation (using GraphPad version 6). Linear regression models were fitted for the avidity of antibody levels in the final visit (May 2018) using the number of infections by microscopy, PCR, or both as the predictor variables. The association of *P. falciparum* carriage in previous months with that of May and their corresponding odds ratio was also determined using Graphpad prism. Statistical significance was determined at *P <*0.05. The relative avidity index (RAI) for Pfs230 IgG, Pfs48/45 IgG was calculated as the ratio of the IgG concentration of the sodium thiocyanate-treated sample to the IgG concentration of the corresponding untreated sample multiplied by 100.

Plasma samples were assigned seropositive for antibodies against an antigen if the concentration of the antibodies in the sample was greater than the average plus two times the standard deviation of that for the negative control sample (pooled malaria-naïve plasma).

## Results

### Clinical Characteristics of the Study Participants

The study recruited non-febrile school children aged from 6 to 12 years residing in the Simiw community in the Central Region of Ghana. A total of 84 children with a median (IQR) age of 9 (8–11) years were recruited in November 2017 and followed until May 2018. A total of 43 of the children were below 10 years old. No significant differences were observed in the median haemoglobin levels (Kruskal–Wallis statistic = 5.250, *P* = 0.1544) and temperature (Kruskal–Wallis statistic = 5.966, test *P* = 0.1133) of the children at all the four-time points (**Table 1**).

**Table 1:** Characteristic of the study participants.

	November 2017	January 2018	March 2018	May 2018	*P*-value
Number of children	84	84	84	84	
Children <10 yrs	43	43	43	43	
Children ≥10 yrs	41	41	41	41	
Hb (g/dl)					
Median	11.35	11.50	11.60	11.60	0.1544
IQR	10.30–12.20	10.70–12.20	10.70–12.30	10.80–12.50	
Temp (^°^C)					
Median	36.70	36.40	36.40	36.40	0.1133
IQR	36.13–36.9	36.3–36.8	36.10–36.7	36.0–36.70	

Hb, Hemoglobin, Temp, Temperature; IQR, Interquartile range; yrs, years.

Asexual parasite prevalence estimated by microscopy was highest in March 2018 (36.9%) and lowest (7.14%) in May 2018, with the highest and lowest median parasite densities detected in May 2018 and January 2018 respectively (**Table 2**). Parasite prevalence estimated by polymerase chain reaction was highest (74.4%) in May and lowest (27.3%) in January. Variation in asexual parasite density was not statistically significant over the four time points (*P* = 0.2303, Kruskal–Wallis statistic = 4.305). None of the samples used in this study tested positive for gametocytes by microscopy (**Table 2**) at any time point. Parasite carriage in May was significantly associated with that of March (Fisher’s exact text, p = 0.0118) with participants who carried parasites in March having odds of 5.3 of being infected in May (**Additional File 1**).

**Table 2:** P*. falciparum* parasite density and prevalence by microscopy and polymerase chain reaction.

	November 2017	January 2018	March 2018	May 2018	*P*-value
Microscopy					
Median PD/µl (IQR)	1040.0	280.0	880.0	1920.0	0.2303
	(300.0–4680)	(160.0–360.0)	(400.0–2440)	(80.0–4330)	
Asexual parasite prevalence (% (n/N))	26.2	8.3	36.9	7.14	
	(22/84)	(7/84)	(31/84)	(6/84)	
Gametocyte prevalence (% (n/N))	0	0	0	0	
	(0/84)	(0/84)	(0/84)	(0/84)	
Polymerase chain reaction					
Total parasite prevalence (% (n/N))	27.3	46.4	42.9	74.4	
	(23/84)	(39/84)	(36/84)	(60/84)	

PD, Parasite density (reported per µl blood); IQR, Interquartile range; n, number of samples confirmed positive for P. falciparum by polymerase chain reaction; N, total number of samples tested for polymerase chain reaction.

### Dynamics of Antibody Responses Against Pfs48/45

**Figure 2:**
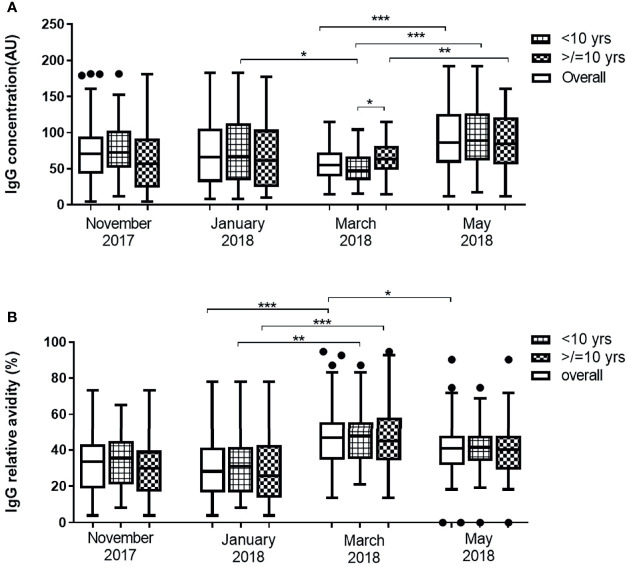
Changes in Pfs48/45 IgG level **(A)** and avidity **(B)** in 43 children <10 years and 41 children ≥10 years over the dry season spanning November 2017 to May 2018. Yrs, years. The extent of statistically significant difference in measurements indicated by *, ** and *** represent P <0.05, P <0.01, and P <0.001 respectively.

The overall median IgG level against Pfs48/45 did not show statistically significant variation from November 2017 to March 2018. In May, however, overall anti-Pfs48/45 IgG levels increased significantly compared to all preceding months of the dry season assessed (p <0.0001). The median Pfs48/45 IgG levels in both the young children (<10 yrs) and older children (≥10 yrs) did not vary significantly from November 2017 to March 2018 except the period from January to March, where IgG levels of the young children decreased significantly (P = 0.867). In May, however, median anti-Pfs48/45 antibody levels increased significantly relative to March levels for both young children (P<0.001) and older children (P<0.01) (**Figure 2A**). Between the two age groups, older children had higher antibody levels than younger children in only March 2018, the second half of the dry season, but statistically the same for the other months assessed.

The median relative avidities of anti-Pfs48/45 IgG in all the study participants (overall and age stratified) did not vary statistically from November to January. In March 2018 however, median relative avidity increased significantly for all children (P <0.001). Transitioning from March to May produced a significant decrease in the overall median relative avidities (P = 0.0292) although it was statistically unchanged for both the young children (P = 0.106) and older children (P = 0.103) (**Figure 2B**) sub-groups. Anti-Pfs4845 antibody levels but not avidity correlated positively (r = 0.282, P = 0.0095) with age in only March, the end of the dry season (**Additional File 1**). Within individuals, both the level and relative avidity of anti-Pfs48/45 IgG varied significantly between the various time points (P <0.0001 for both) (**Additional File 1**)

### Dynamics of Antibody Responses Against Pfs230

**Figure 3:**
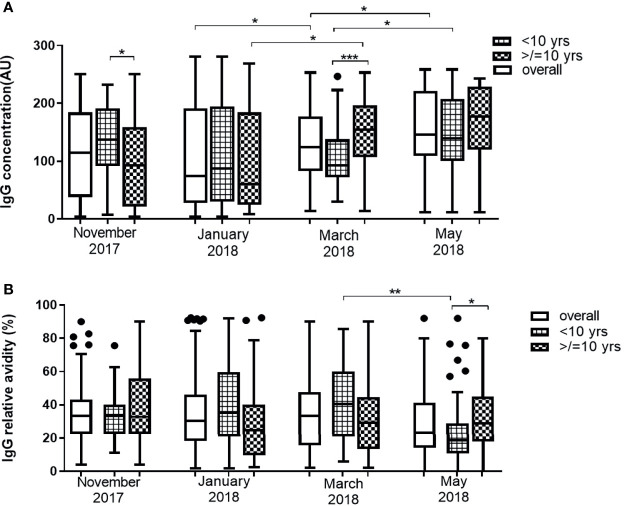
Changes in Pfs230 IgG level **(A)** and avidity **(B)** in 43 children <10 years and 41 children ≥10 years over the dry season spanning November 2017 to May 2018. The extent of statistically significant difference in measurements indicated by *, ** and *** represent P <0.05, P <0.01, and P <0.001 respectively.

The median levels of anti-Pfs230 antibodies of the overall participants significantly increased in March (P = 0.001) and in May (P = 0.0125). In younger children, median IgG levels against Pfs230 increased significantly in only May (P = 0.014). In older children, however, median anti-Pfs230 antibody levels had a statistically significant increase in March (P = 0.002) and remained the same in May (P = 0.250). Median antibody levels were higher for young children than older children in November 2017, the beginning of the first half of the dry season (P = 0.036), while the reverse occurred in March 2018 (P = 0.0007), close to the end of the second half of the dry season (**Figure 3A**). There were no statistically significant differences in the median relative avidities for the participants as a whole across the four time points (P = 0.0673). The relative avidity of anti-Pfs230 IgG in older children (>10 yrs) did not show any statistically significant variations (P = 0.325) over the duration of the study (**Figure 3B**). In younger children (<10 yrs) however, the median relative avidities of anti-Pfs230 IgG dropped significantly in May (P <0.001), to values lower than recorded in the older children (**Figure 3B**). Similar to Pfs48/45, anti-Pfs230 antibody levels correlated positively with age (r = 0.358, P = 0.008) but the relative avidity did not. In May however, both the levels and relative avidities of IgG against Pfs230 were positively correlated (r = 0.225, P = 0.0397 and r = 0.236, P = 0.0305, respectively) with age. The anti-Pfs230 antibody levels and relative avidities also varied significantly within individual participants (P = 0.0049 and P = 0.026, respectively) (**Additional File 1**)

### Seroprevalence of Antibodies Against Pfs230 and Pfs48/45

**Figure 4:**
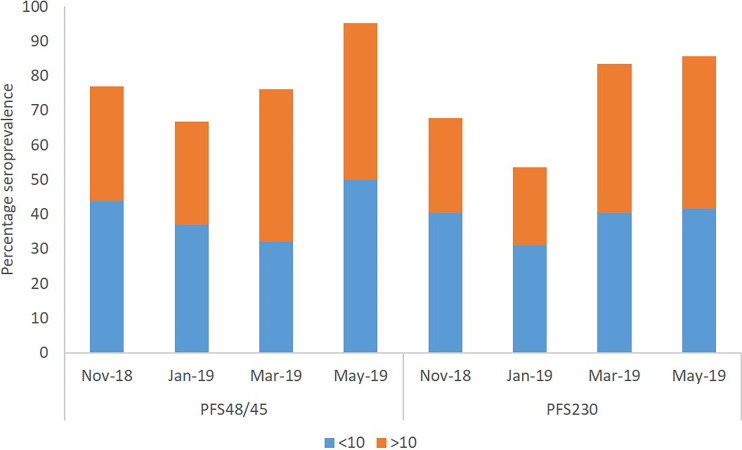
Seroprevalence of IgG antibodies against Pfs230 and Pfs48/45. Immune responses of participants were considered seropositive when their antibody concentrations were higher than that of the average plus twice the standard deviation of the negative control sample’s concentration.

Percentage seroprevalence of IgG antibodies against both Pfs48/5 and Pfs230 of all the participants had a similar trend, decreasing from the beginning of the dry season (November 2018) to the middle of the dry season (January) after which it increased again by the end of the dry season (March) and the early rainy season (May) (**Figure 4**). The observed trend of overall seroprevalence for both antigens was similar to that of median parasite densities of participants. The percentage seroprevalence of both the younger children and older children categories followed the same trend as the overall participants. In November and January, younger children had a higher seroprevalence of IgG against both antigens than older children. In March and May, however, older children had a higher seroprevalence of antibodies against Pfs230 than the younger children. For anti-Pfs48/45 however, older children had a higher Percentage seroprevalence than younger children in only March (**Figure 4**).

### Dynamics of Antibody Responses in the Presence or Absence of Active *P. falciparum* Infection

**Figure 5:**
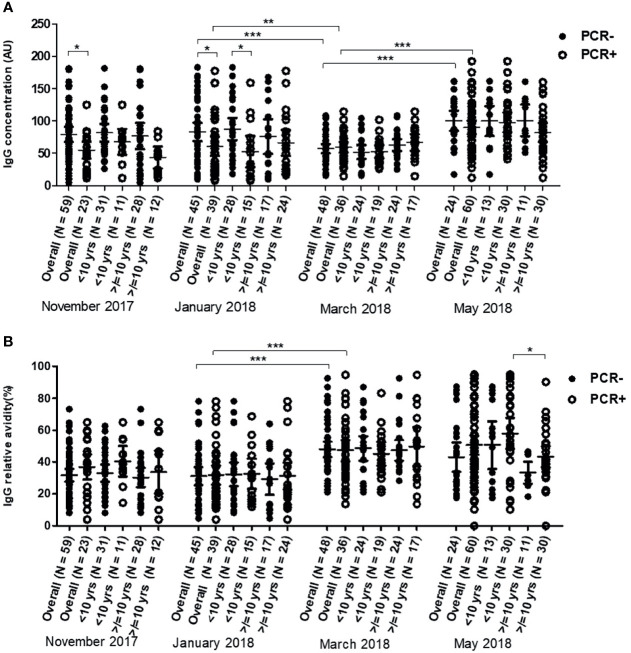
Variations in of anti-Pfs48/45 IgG levels **(A)** and Relative avidities **(B)** in young children (<10 years) and older children (≥10 years) with (positive polymerase chain reaction test result) and without (negative-polymerase chain reaction test result) active *P. falciparum* infections over the dry season from November 2017 to May 2018. The number above each of the aligned dot plots represents the number of individuals in the group. The extent of statistically significant difference in measurements indicated by *, ** and *** represent P <0.05, P <0.01, and P <0.001 respectively.

In the overall dataset, anti-Pfs48/45 IgG levels were higher in children without active infections in November (P = 0.027) and January (p = 0.019). In March however, the IgG levels in children without active infections dropped to similar levels as children with active infections, whose IgG levels remained unchanged from November to March. In May, the early parts of the rainy season anti-Pfs48/45 IgG in both children with and without active infections rose significantly relative to March levels (P <0.001 for both months) (**Figure 5A**).

Children with and without active infections had similar relative anti-Pfs48/45 IgG avidity at all assessed time points. The relative avidities were unchanged between November and January but increased significantly in March (P <0.001) and remained at the same level in May (**Figure 5B**) for both children with and without active infection.

Each of the two age groups (children <10 and ≥10 yrs) were sub-grouped into those with active infections (parasites detected by polymerase chain reaction, PCR+) and those without active infections (no parasites detected by polymerase chain reaction, PCR−) and their anti-Pfs48/45 and anti-Pfs230 IgG antibody levels and avidities compared. It was observed that the antibody responses (both IgG levels and avidities) in the two groups of children (with and without active infection) were similar throughout the study period except in January when the Pfs48/45 IgG levels of young children without active infections was higher than their counterparts with active infections (*P* = 0.0112) (**Figure 5A**). In May, when younger children without an active infection had higher relative avidity (P = 0.019) than older children with active infection (**Figure 5B**).

**Figure 6:**
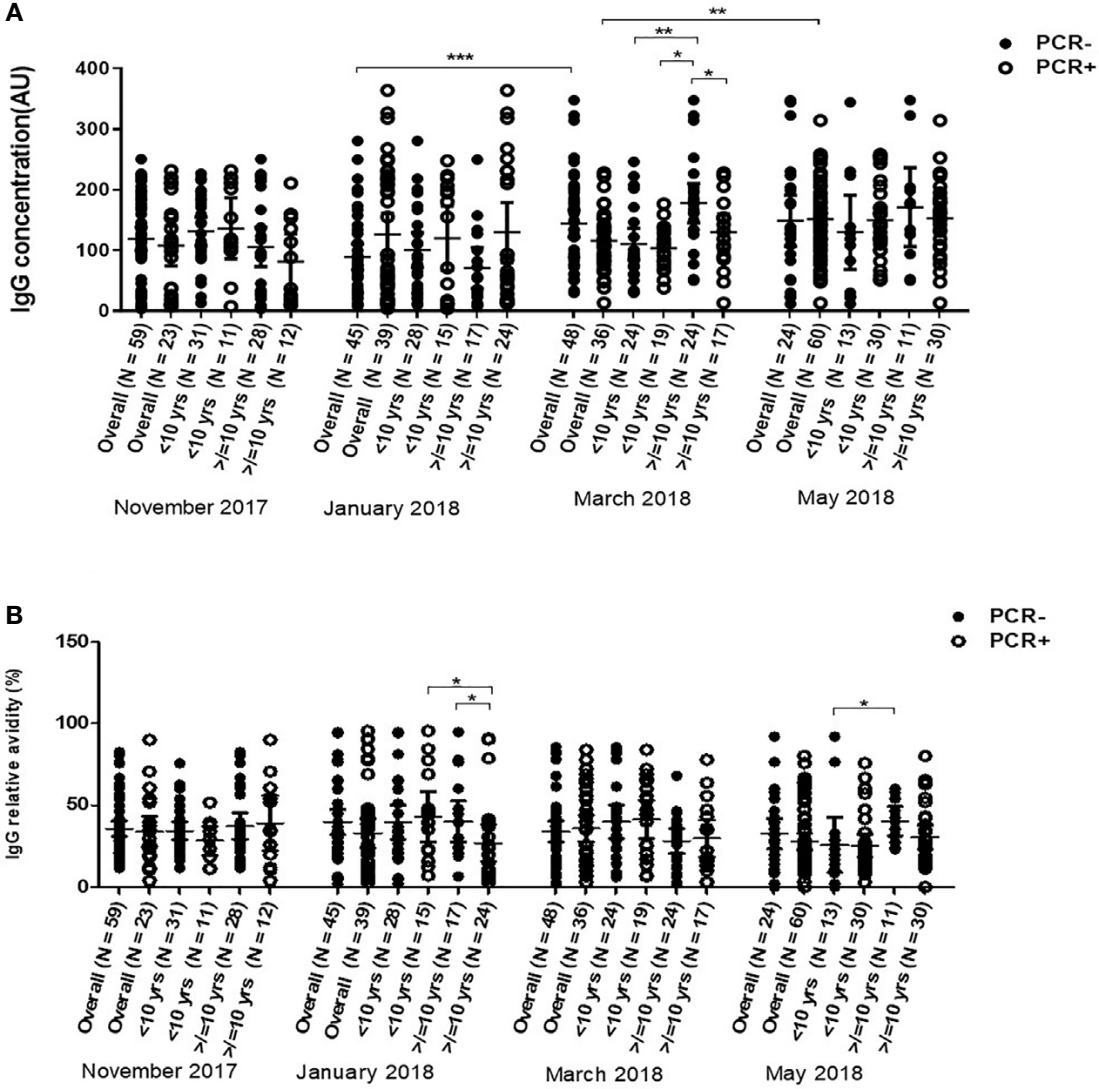
Variations in of anti-Pfs230 IgG levels **(A)** and Relative avidities **(B)** in young children (<10 years) and older children (≥10 years) with (positive polymerase chain reaction test result) and without (negative-polymerase chain reaction test result) active *P. falciparum* infections over the dry season from November 2017 to May 2018. The number above each of the aligned dot plots represents the number of individuals in the group. The extent of statistically significant difference in measurements indicated by *, ** and *** represent P <0.05, P <0.01, and P <0.001 respectively.

Overall, children with active infections had similar anti-Pfs230 IgG levels as those without active infections at all-time points. Anti-Pfs230 IgG levels however increased in children without active infection from January, mid dry season, to March which is towards the end of the dry season. Children with active infections in May had higher anti-Pfs230 IgG levels than in March (P = 0.0041). Children with and without active infections had similar median relative avidities at all-time points in all children as a whole or in the two age categories (**Figure 6B**). In March, older children (≥10 yrs) without active infections had significantly higher anti-Pfs230 IgG levels than their age mates with active infections (P = 0.040) and young children (<10 yrs) with (*P* = 0.0003)) or without active infections (*P* = 0.00135) (**Figure 6A**).

In May, the median relative avidity of Pfs230 IgG in older children (≥10 yrs) without active infections was significantly higher (*P* = 0.0263) than in younger children <10 yrs with active infections (**Figure 6B**). Older children with active infection had significantly lower relative avidity of anti-Pfs230 IgG compared to their age mates without active infections (P = 0.0378) or young children with active infections (P = 0.0315) in January. In May, however, the median relative avidity of older children with active infections rose to that of older children without active infection whose median relative avidity was significantly higher than young children without active infection (**Figure 6B**). The individual antibody levels and relative avidities used in this section can be found in **Additional File 1**. Regressional analyses showed that the number of times an individual tested positive for parasites by PCR or microscopy were not significant predictors of the antibody levels and avidities against the two antigens. Consequently, there was no statistical difference between the number of times a person had been infected during our visits and the anti-Pfs230 parasite and anti-Pfs48/45 antibody levels and avidity (**Additional Files 1 and 2**).

### Association Between IgG Responses Against Pfs48/45 and Pfs230

In November, no correlation was identified between the measured IgG levels and avidity against Pfs48/45. Moderate and positive correlations were identified in January which changed to a weak inverse correlation in both March and May. All the correlations identified other than in November were significant.

Significant negative correlations existed between IgG levels and avidity against Pfs230 throughout the study period. The correlation was strong in the first half of the study period but weak towards the peak season (in the second half of the study period) (**Table 3**).

**Table 3:** Correlation between IgG level and avidity against Pfs230, Pfs48/45.

	Pfs48/45 IgG (level *vs* avidity)	Pfs230 IgG (level *vs* avidity)
Nov.2017	R = 0.0251,	r = −0.5287,
	*P* = 0.7999	*P <*0.0001
Jan.2018	r = 0.4175,	r = −0.6911,
	*P <*0.0001	*P <*0.0001
Mar.2018	r = −0.2177,	r = −0.1824,
	*P* = 0.0360	*P* = 0.0800
May.2018	r = −0.2609,	r = −0.2405,
	*P* = 0.0081	*P* = 0.0149

r, correlation coefficient; P, p-value; vs, versus.

## Discussion

Although there are numerous reports on the development of immunity against the disease-causing asexual *P. falciparum* parasite, relatively few reports have characterized the development of immunity against the sexual stages of *P. falciparum*. However, this knowledge can help improve the development of transmission-blocking vaccines to reduce malaria transmission. This study, for the first time, characterizes both quantity and quality of naturally acquired antibodies to both transmission-blocking vaccine candidates, Pfs230 and Pfs48/45 over the course of the dry season into the early rainy season.

This study was conducted at the end of the peak malaria season through to the beginning of the subsequent peak season, a period where there is a paucity of mosquito vectors (Amoah et al., 2018; Amoah et al., 2019a). The persistence of *P. falciparum* throughout the study period suggests that the possibly few mosquito vectors circulating during the off-peak season were able to sustain malaria transmission within the community. Carriage of *P. falciparum* can result in the development of anemia (Doolan et al., 2009; Magombedze et al., 2018; Epopa et al., 2019) however, most of the children in this study, despite harboring malaria had normal hemoglobin levels. This could however be because asymptomatic infections predominantly contain low parasite densities, which are less likely to result in anemia as compared with high parasite density infections (Doolan et al., 2009; Magombedze et al., 2018; Epopa et al., 2019).

IgG levels against both Pfs230 and Pfs48/45 were highest in May which is the beginning of the peak season. This observation was somewhat similar to our previous observation of higher anti-Pfs230 IgG levels in the peak season compared to the off-peak season and suggested an increased exposure to gametocytes compared to the preceding months assessed in this study (Amoah et al., 2019a). Although no gametocytes were identified by microscopy over the course of the study, the persistent replication of asexual parasites during the erythrocytic life cycle of the parasite is known to result in the production of low densities of gametocytes that are detectable by techniques with higher sensitivities than microscopy (Alano, 2007; Reuling et al., 2018). Prolonged exposure to submicroscopic gametocyte densities has previously been identified to induced antibody responses (Haldar & Mohandas, 2009; Amoah et al., 2019a; Muthui et al., 2020). A previous report from a similarly high malaria transmission setting of southern Ghana identified a higher prevalence of submicroscopic gametocytes densities during the off-peak season compared with the peak season (Ayanful-Torgby et al., 2018).

The trends in the relative avidity of IgG responses against Pfs48/45 antigen observed in both young and older children (<10 yrs or ≥10 yrs) were similar to the trend of asexual parasite prevalence estimated by microscopy. This is similar to observations made for the relative avidities of antibodies against asexual parasite antigens, which are low in high transmission settings due to frequent exposure to highly diverse parasite strains (Abagna et al., 2018). However, for most malaria infections, gametocyte densities are significantly lower than those of the asexual parasite (Alano, 2007; Reuling et al., 2018) and as such the lower numbers and diversity of gametocytes in the high transmission setting may be responsible for the inverse relationship between the relative avidity of antibody responses against sexual stage antigens relative to asexual stage antigens. The similarity of the pattern of the percentage seroprevalence and median parasite densities suggests that the level of antibodies produced was proportional to the parasitemia of infection.

Participants, especially older children (10–12 years old), with active infections generally showed a trend of lower antibody levels against both sexual stage antigens than those without active infection. This observation could be due to the concomitant large amounts of antibodies produced in response to recent past exposure to asexual and sexual stage parasites that are currently protecting against parasite infection. At the onset of the peak season (in May) the relative avidity of Pfs48/45 IgG in the young children (<10 years) with active infections were significantly higher than that of the older children (≥10 years) with and without active infection. A possible explanation for this could be the increase in exposure to low-density gametocytes in younger children relative to older children as gametocyte carriage has been suggested to be more prevalent in young children relative to the older population (Ouedraogo et al., 2007). The relative IgG avidity against Pfs230 at a time point close to peak malaria season was observed to be significantly higher in older children (≥10 years) than in younger children (<10 years), suggesting that age could have an impact on the avidity. This increase in IgG antibody avidity with the increase in age could be due to increased exposure to the same antigen and hence the tendency of IgG avidity to develop or have matured better in adulthood than in childhood as recently reported (Tassi Yunga et al., 2021). Lack of an association between the number of times a person had been infected with parasites during the follow-ups and antibody levels or avidities was not surprising as all microscopic infections determined contained only asexual parasites. Furthermore, most of the submicroscopic infections could contain only asexual forms of the parasite or gametocytes at thresholds not high enough to stimulate high levels of immune response.

The observed correlation between the level and relative avidity being positive for Pfs48/45 IgG but negative for that of Pfs230 IgG in November and January (early parts of the off-peak malaria season), could be suggestive of differences in decay kinetics associated with dynamic conformational flexibility (Huber, 1979; Amaral et al., 2017) of antibodies against both antigens, despite biological relatedness existing between both antigens and a half-life of about three months having been determined for their antibodies. The negative correlation observed between IgG levels and avidities against both Pfs230 and Pfs48/45 in the second half of the study period, towards the peak malaria season, could be due to the effect of an increase in the frequency and persistence of high diversity infections on IgG responses (IgG avidities and levels) towards the peak season relative to the beginning as well as during the off-peak season (Sondo et al., 2020). Consistent infections have also been suggested to impair antibody affinity maturation in germinal centers and result in the production of antibodies with low avidity (Ssewanyana et al., 2017). An earlier study conducted in southern Ghana also reported an inverse relation between Pfs48/45 IgG levels and avidity (Amoah et al., 2019a).

### Study Limitations

The quality of the antibodies against an antigen is determined by higher binding affinity as well as functionality. This study did not determine the functionality of the antibodies beyond determining the relative avidity index. Functional assays such as antibody-mediated complement lysis or Opsonic phagocytosis are important measures of antibody functionality. We could however not perform these assays due to limited funding. Furthermore, submicroscopic gametocyte densities, as well as the entomological inoculation rate during the period of the study, were also not determined. These could have provided additional measures of exposure rates as well as the risk of parasite infection due to gametocytes whose surface antigens were studied in the current study. However, studies have identified submicroscopic gametocyte densities in samples that were classified as negative for gametocytes by microscopy (Shekalaghe et al., 2007; Lamptey et al., 2018) as well as have identified a very low prevalence of mosquito vectors in the off-peak malaria season (Magombedze et al., 2018; Epopa et al., 2019).

## Conclusion

IgG responses against Pfs48/45 and Pfs230 antigens were higher at the end of the off-peak season compared to the beginning. The relative avidities of IgG responses against Pfs230 and Pfs48/45 were inversely correlated to their IgG levels from the middle to the end of the off-peak season.

## Data Availability Statement

The datasets used and/or analyzed during the current study are included in the article or **Supplementary Material**.

## Ethics Statement

The studies involving human participants were reviewed and approved by The Institutional Review Board (IRB) of the Noguchi Memorial Institute for Medical Research (NMIMR). Written informed consent to participate in this study was provided by the participant’s legal guardian/next of kin.

## Author Contributions

LA designed the study. LA, FA, and FB performed the statistical analysis. ES and FB performed the experiments. EO and ES collected the samples. LA, D-OY, FA, EO, ES, and FB wrote and revised the final manuscript. All authors contributed to the article and approved the submitted version.

## Funding

The principal researcher funded this work from personal funds.

## Conflict of Interest

The authors declare that the research was conducted in the absence of any commercial or financial relationships that could be construed as a potential conflict of interest.

## Publisher’s Note

All claims expressed in this article are solely those of the authors and do not necessarily represent those of their affiliated organizations, or those of the publisher, the editors and the reviewers. Any product that may be evaluated in this article, or claim that may be made by its manufacturer, is not guaranteed or endorsed by the publisher.

## Abbreviations

IgG, immunoglobulin G**;** PCR, polymerase chain reaction**;** HB, hemoglobin**;** aa, Amino Acid; DNA, Deoxyribonucleic acid; wConc, weighted concentration; PD, parasite density; RAI, relative avidity index; yrs, years.
