# Supplementary figures and images for: Profiling the Quality and Quantity of Naturally Induced Antibody Responses Against Pfs230 and Pfs48/45 Among Non-Febrile Children Living in Southern Ghana: A Longitudinal Study
Source: Front Cell Infect Microbiol. 2021 Nov 25;11:770821. doi: 10.3389/fcimb.2021.770821 (PMC8656302; doi:10.3389/fcimb.2021.770821)

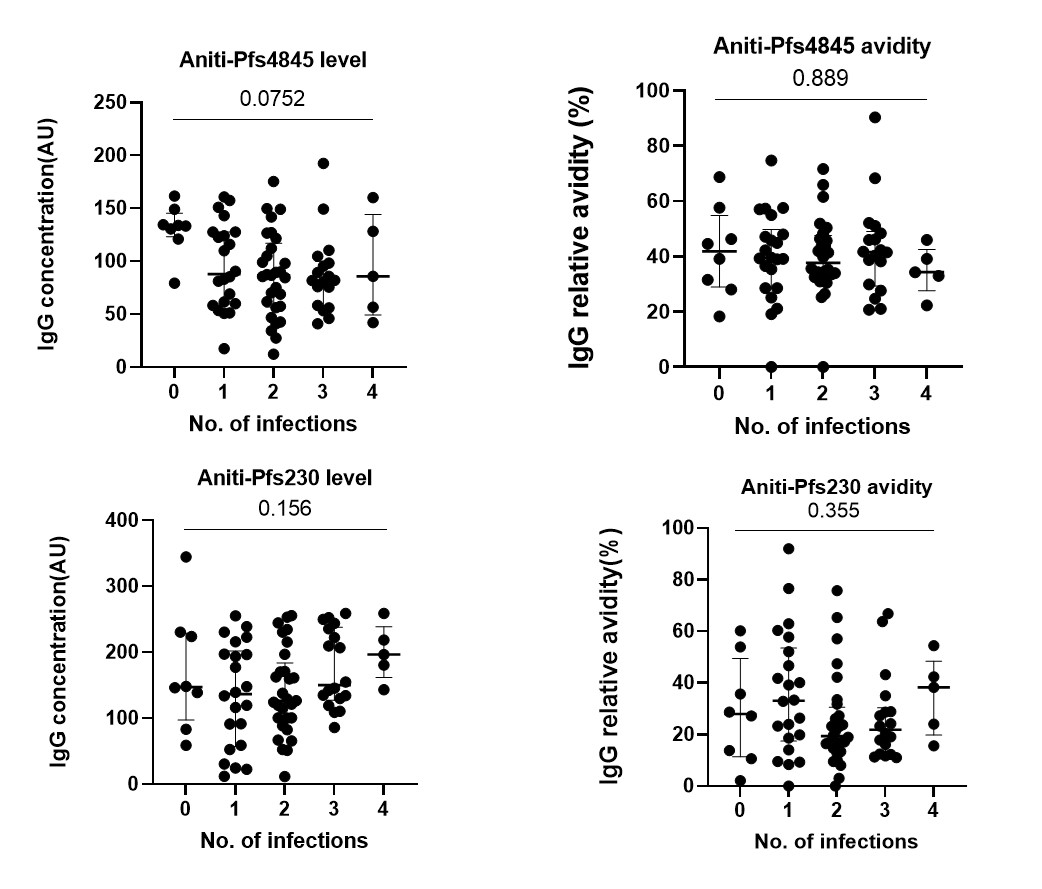

Supplement: Supplementary file 1 [file Image_1.jpeg]
